# Supplementary material for: Subchronic Exposure to Polystyrene Microplastic Differently Affects Redox Balance in the Anterior and Posterior Intestine of Sparus aurata
Source: Animals (Basel). 2023 Feb 9;13(4):606. doi: 10.3390/ani13040606 (PMC9951662; doi:10.3390/ani13040606)
Supplement: Supplementary file 1 [file animals-13-00606-s001.zip › animals-2148960-Table S1.pdf]

Table S1. Fish feed composition.

Gross Energy (MJ/kg)	18,75
Digestible energy (MJ/kg)	16,67
Crude Fat (%)	17,90
Crude Protein (%)	42,34
Digestible proteins (%)	36,14
Fish Protein (%)	8,24
Animal Protein (%)	27,34
Fish protein/Total protein (%)	19,45
Digestible protein/Digestible energy (mg/kJ)	21,68
Animal protein/Total protein (%)	64,59
Fiber (%)	2,81
Uninzoted extracts (%)	18,05
Starch (%)	7,71
Non-starch polysaccharides (%)	13,15
Dry Matter (%)	88,31
Starch In feed (%)	7,71
Ash in feed (%)	7,21
Fiber in Feed (%)	2,81
Ala (%)	2,78
Arg (%)	2,71
Asp (%)	3,64
Glu (%)	5,93
Gly (%)	3,40
His (%)	1,11
Iso (%)	1,55
Leu (%)	3,28
Lys (%)	2,54
Met (%)	1,04
Phe (%)	1,97
Pro (%)	2,55
Ser (%)	2,20
Thr (%)	1,75
Tyr (%)	1,24
Trp (%)	0,39
Val (%)	2,17
Taurine (%)	0,46
Vit. D (I.U./Kg)	2100
Vit. A (I.U./Kg)	8000
Vit. E (mg/Kg)	389,72
Inositol (mg/Kg)	192,83
Niacin (mg/Kg)	229,17

Pantothenic acid (mg/Kg)	88,97
Vit. B2 (mg/Kg)	33,98
Vit. B1 (mg/Kg)	34,31
Vit. B6 (mg/Kg)	27,87
Folic acid (mg/Kg)	7,93
Vit. K (mg/Kg)	6,56
Biotin (mg/Kg)	1,12
Vit. B12 (mg/Kg)	0,06
Choline (mg/Kg)	1698,71
Vit. C (mg/Kg)	250,25
Calcium (mg/Kg)	1,60
Cobalt (mg/Kg)	0,34
Copper (mg/Kg)	45,64
Iron (mg/Kg)	577,18
Magnesium (mg/Kg)	0,18
Manganese (mg/Kg)	47,47
Molybdate (mg/Kg)	0,54
Nichel (mg/Kg)	1,97
Phosphorus (mg/Kg)	1,17
Potassium (mg/Kg)	0,90
Sodium (mg/Kg)	0,21
Selenium (mg/Kg)	0,62
Sulfur (mg/Kg)	0,56
Zinc (mg/Kg)	83,05
Tot myristic acid (C14:0, %)	0,18
Tot pentadecanoic acid (C15:0, %)	0,02
Tot palmitic acid (C16:0, %)	1,36
Tot palmitoleic acid (C16:1, %)	0,27
Tot heptadecanoic acid(C17:0, %)	0,02
Tot heptadecenoic acid (C17:1, %)	0,02
Tot stearic acid (C18:0, %)	0,42
Tot oleic acid (C18:1 n-9, %)	7,75
Tot vaccenic acid (C18:1 n-7, %)	0,23
Tot linoleic acid (C18:2 n-6, LA, %)	3,11
Tot α-linolenic acid (C18:3 n-3, ALA %)	1,01
Tot γ-linolenic acid (C18:3 n-6, %)	0,02
Tot octadecatrienoic acid (C18:4 n-3, %)	0,06
Tot arachidic acid (C20:0, %)	0,07
Tot eicosenoic acid (C20:1 n-9, %)	0,32
Tot eicosadienoic acid (C20:2 n-6, %)	0,05
Tot eicosatrienoic acid (C20:3 n-6, %)	0,01
Tot eicosatrienoic acid (C20:3 n-3, %)	0,01

Tot arachidonic acid (C20:4 n-6, %)	0,04
Tot eicosatetraenoic acid (C20:4 n-3, %)	0,03
Tot eicosapentaenoic acid (C20:5 n-3, EPA, %)	0,29
Tot behenic acid (C22:0, %)	0,06
Tot cetoleic acid (C22:1 n-11, %)	0,15
Tot erucic acid (C22:1 n-9, %)	0,04
Tot docosadienoic acid (C22:2 n-6, %)	0,02
Tot docosapentaenoic acid (C22:5 n-3, DPA, %)	0,06
Tot docosahexanoic acid (C22:6 n-3, DHA, %)	0,40
Tot lignoceric acid (C24:0, %)	0,01
Tot tetracosahexaenoic acid (C24:1 n-9, %)	0,02
Tot pentacosanoic acid (C25:0, %)	0,01
Tot Omega 3 (%)	2,09
Tot Omega 6 (%)	3,94
Omega 3/Omega 6 (ratio)	0,53

Percentage values are expressed on total feed.
