# Supplementary material for: Subchronic Exposure to Polystyrene Microplastic Differently Affects Redox Balance in the Anterior and Posterior Intestine of Sparus aurata
Source: Animals (Basel). 2023 Feb 9;13(4):606. doi: 10.3390/ani13040606 (PMC9951662; doi:10.3390/ani13040606)
Supplement: Supplementary file 1 [file animals-13-00606-s001.zip › animals-2148960-Table S2.pdf]

Table S2. Primer sequences used for Real Time-PCR analysis.

Gene	Gene bank ID	Primer Forward (5'→3')	Primer Reverse (5'→3')
Rps18	AM490061	AGGGTGTTGGCAGACGTTAC	CGCTCAACCTCCTCATCAGT
Nrf2	Unpublished	GTTCAGTCGGTGCTTTGACA	CTCTGATGTGCGTCTCTCCA
Superoxide dismutase	AJ937872	CCATGGTAAGAATCATGGCGG	CGTGGATCACCATGGTTCTG
Catalase	FG264808	TTCCCGTCCTTCATTCACTC	CTCCAGAAGTCCCACACCAT
Glutathione reductase	AJ937873	CAAAGCGCAGTGTGATTGTGG	CCACTCCGGAGTTTTGCATTTC
Heat-shock protein 70	EU805481	AATGTTCTGCGCATCATCAA	GCCTCCACCAAGATCAAAGA
Heat-shock protein 90	DQ524994.1	GTGGATTCTGAGGACCTGCC	GAGAGTCTTCGTGGATGCCC
